# Genus *Veronica*—Antioxidant, Cytotoxic and Antibacterial Activity of Phenolic Compounds from Wild and Cultivated Species

**DOI:** 10.3390/antiox14111308

**Published:** 2025-10-30

**Authors:** Ivana Vrca, Antonija Mikrut, Željana Fredotović, Karla Akrap, Dario Kremer, Stjepan Orhanović, Katarina Bačić, Valerija Dunkić, Marija Nazlić

**Affiliations:** 1 Faculty of Science, University of Split, Ruđera Boškovića 33, 21000 Split, Croatia; ivrca@pmfst.hr (I.V.); zfredotov@pmfst.hr (Ž.F.); kakrap@pmfst.hr (K.A.); stipe@pmfst.hr (S.O.); kbacic@pmfst.hr (K.B.); dunkic@pmfst.hr (V.D.); 2 Teaching Institute for Public Health Split, Vukovarska 46, 21000 Split, Croatia; mikrut.antonija@gmail.com; 3 Faculty of Agriculture, University of Zagreb, Svetošimunska cesta 25, 10000 Zagreb, Croatia; dkremer@agr.hr

**Keywords:** *Veronica*, speedwells, phenolic compounds, LC-QTOF, antibacterial activity, disk diffusion, antioxidant activity, DPPH, ORAC, cytotoxic activity

## Abstract

(1) Background: The conservation of plant resources is important because many wild plant populations are threatened by various influences. Growing plants from seeds is one way to ensure their survival. Comparing the biological potential of extracts between plants cultivated from seeds and wild plants provides information about their specialized metabolites. For this reason, this study compared the biological potential of phenolic extracts from four wild-collected species of the genus *Veronica* and the same four cultivated species. The studied species of the genus *Veronica* are *V. anagallis-aquatica* L., *V. persica* Poir., *V. polita* Fr. and *V. hederifolia* L. (2) Methods: The phenolic composition was investigated with LC-QTOF (Liquid Chromatography-Quadrupole Time-of-Flight). The main methods for biological activities were as follows: ORAC (Oxygen Radical Absorbance Capacity) and DPPH (2,2-diphenyl-1-picrylhydrazyl) radical for antioxidant activity, the disk diffusion test for antibacterial activity and the MTS test of cytotoxic activity. (3) Results: The major compound in all extracts was apigenin. Cultivated species showed higher antioxidative activity. Phenolic compounds isolated from the *V. anagallis-aquatica* species showed the highest cytotoxic effect on all tested lines. The extracts showed antibacterial activity on three bacterial strains: *Streptococcus pyogenes*, *Listeria monocytogenes* and *L. innocua.* Extracts of *V. anagallis-aquatica* showed the highest antibacterial activity, both from the natural habitat and cultivated habitat. (4) Conclusions: A comparison of the different activities tested for phenolic extracts between wild and cultivated species of the genus *Veronica* showed that cultivated species also have significant biological activity and are suitable for further research and applications.

## 1. Introduction

The genus *Veronica* is the largest genus in the Plantaginaceae family that is very well adapted to various habitats. Plants from this genus are used in traditional medicine, mainly for lung and respiratory diseases [1]. Previous research on specialized metabolites isolated from many species of the genus *Veronica* included both free volatile compounds [2] and bound volatile compounds such as iridoids, saponins and phenolic components [1,3,4,5,6]. Based on the study of the phenolic profiles of the species *Veronica montana*, *V. polita* and *V. spuria*, the authors concluded that the studied species can be considered as good sources of phenolic compounds for industrial or pharmacological applications [7]. The study of phenolic compounds from plants is important due to their potent antioxidant properties, the effect of which is evident in the prevention of various diseases associated with oxidative stress [8,9]. Earlier studies on the antioxidant and antimicrobial activity of phenolic extracts from the genus *Veronica* were carried out on the species *V. officinalis* L., *V. teucrium* L. and *V. orchidea* Crantz [2]. In addition, the antioxidant effect of phenols isolated from *Veronica jacquini*, *V. teucrium* and *V. urticifolia* Jacq was investigated *in vivo* using a hepatic antioxidant system assay in rats [10,11]. Natural antioxidants may be more effective than synthetically produced compounds in preventing the effects associated with oxidative stress, as synergistic interactions between plant isolates may enhance the antioxidant effect [12,13]. This is precisely why it is important to conserve plant resources, especially plant species whose products play an important ecological role in their habitats. Among the cytotoxic activities of extracts of the genus *Veronica*, we emphasize the significant cytotoxic activity of methanolic and aqueous extracts of the species *V. persica* and *V. polita* against mouse melanoma cells [1]. As amphipathic molecules, phenolic compounds also have an antibacterial effect by interacting with proteins and bacterial cell walls and also act on DNA by damaging bacterial DNA and altering its stability [14]. Antibacterial resistance is a major problem, so it is necessary to find alternative therapeutic options [15]. Herbs can be used in the form of plant extracts or as their active components. The use of *Veronica* species in ethnobotany is also well known, which is another reason demonstrating the importance of their conservation. These and many other species are threatened by human intervention in nature, the conversion of land into agricultural or buildings and global warming [16]. This is why in this research selected species were cultivated in the botanical garden, and their extracts were tested. The species of the genus *Veronica* (speedwell) selected for this research are *V. anagallis-aquatica* L. (water speedwell), *V. persica* Por. (bird’s-eye speedwell, common speedwell, Persian speedwell), *V. polita* Fr. (gray speedwell) and *V. hederifolia* L. (ivy-leaved speedwell). All four species are medicinal plants [1,4], which emphasizes the importance of their conservation. The aim of this study was to test the cytotoxic, antioxidant and antimicrobial potential of phenolic extracts from four wild and four cultivated species of the genus *Veronica* and compare the results to provide conclusions about similarities or differences in the specialized metabolites’ content and activity. This current study is a continuation of our team’s previous work, so to allow for comparison, the results from our previous work, Vrca et al., have been added to the tables for phenolic compounds and antioxidant activity [17].

## 2. Materials and Methods

### 2.1. Cultivation and Drying of Veronica Species

The cultivation of four *Veronica* species from seed was carried out in 2024 in the Department of Ornamental Plants, Landscape Architecture and Garden Art, Faculty of Agriculture, University of Zagreb, Zagreb, Croatia. Four *Veronica* species were *V. anagallis-aquatica* L., *V. hederifolia* L., *V. persica* Poir. and *V. polita* Fr. Seeds of *Veronica* species were sown in pot using potting substrate no. 2 (Klasmann-Deilmann GmBH, Geste, Germany). Three weeks after germination, 1–3 seedlings per pot were transplanted using the same substrate. When the plants were at flowering stage, stems with leaves and flowers were cut off and air dried at 25 °C in dark place for 15 days. The voucher specimens for the cultivated species were deposited in the herbarium of the Laboratory of Botany (HPMF-HR), Faculty of Science, University of Split, Croatia, under the designation CROVeS-01-2024 to CROVeS-04-2024. Besides cultivated species, species from natural habitat were investigated. Information about locality and herbarium is presented in Table 1.

### 2.2. Reagents and Standards for LC/MS-MS and Other Analysis

Formic acid was obtained from Prolabo (VWR International, Rosny-sous-Bois, France), while acetonitrile (HPLC-grade) was obtained from Merck KgA (Darmstadt, Germany) for LC-MS/MS analysis. The pure phenolic components were obtained from Sigma-Aldrich (St. Louis, MO, USA). For cytotoxic activity we used DMEM (Dulbecco’s Modified Eagle Medium) High Glucose (4.5 g/L) with L-Glutamine (Capricorn Scientific GmbH, Ebsdorfergrund, Germany). The CellTiter 96^®^ AQueous One Solution Reagent (Promega, Madison, WI, USA) contains a tetrazolium compound [3-(4,5-dimethylthiazol-2-yl)-5-(3-carboxymethoxyphenyl)-2-(4-sulfophenyl)-2H-tetrazolium, inner salt; MTS] and an electron coupling reagent (phenazine ethosulfate; PES). For the antibacterial activity we used Mastdiscs AST (Mast Group Ltd., Bootle, UK). For the antioxidant activity we used 96-well black polystyrene microtiter plates (Porvair Sciences, Leatherhead, UK), 96-well transparent polystyrene microtiter plates (Porvair Sciences, Leatherhead, UK), 2,2′-Azobis(2-methyl-propionamidine) dihydrochloride (AAPH, Acros Organics, Geel, Belgium), Trolox (Sigma–Aldrich, St. Louis, MO, USA) and methanol (Kemika, Zagreb, Croatia).

### 2.3. Plant Preparation and Extraction

Before the extraction of phenolic components present in different extracts of the above-mentioned *Veronica* species, the dried plant material was milled to a fine powder using a coffee grinding machine. The powdered plant material (0.5 g, final grain size 40 µm) was extracted by maceration at room temperature in the dark with 80% ethanol (*v*/*v*) using as a solvent for 72 h, with a ratio of 1:50 (*w*/*v*) as detailed in Vrca et al. [17]. The extracts were then centrifuged and filtered through blue ribbon filter paper. Afterwards, the extracts were then rotary evaporated at 40–60 °C using a Buche rotavapor R-200 (Flawil, Switzerland) to remove ethanol, while aqueous parts of extracts were lyophilized using a Freeze-dryer Alpha 1–4 LSCplus (Osterode am Harz, Germany). Extraction yield was expressed in mg of lyophilized extract/g of dried plant considering the different extraction yield for each species. The obtained lyophilized dry material was then dissolved in ultrapure water (Milli-Q water made with NIROSTA water purifier model NIRO-VV-UV-UF, Osijek, Croatia) at a stock concentration of 10 mg/mL for testing the biological activity. Acceptance criterion for ultrapure water was ≥18.0 MΩ·cm at 25 °C.

### 2.4. Chemical Identification of Phenolic Components

For chemical identification of phenolic components by LC-MS/MS (liquid chromatography–mass spectrometry) analysis, the different *Veronica* extracts (from natural habitats and cultivated) were prepared and analyzed according to the method by Vrca et al. [17]. A SCIEX—Triple TOF 6600+ mass spectrometer (SCIEX, Marlborough, MA, USA) coupled to a liquid chromatography system (SCIEX—Ex-ionLC, Marlborough, MA, USA.) with a binary pump, as well as a Phenomenex Kinetex Core–Shell 2.6 µm C18 100 Å, 100 × 2.1 mm column, thermostated at 40 °C, were used for LC-MS/MS analysis; 0.1% *v*/*v* formic acid in water (A) and acetonitrile (B) were the solvents, and the flow rate was 0.3 mL/min, while the injection volume was 5 µL. The gradient program was the same as in Vrca et al. [17]. The ion spray voltage was set at −4500 V in the negative ESI mode. Mass spectrometer was calibrated using Sciex ESI negative calibration solution introduced every five samples analyzed. The compounds of interest contained in the sample extracts were characterized by mass spectra and retention times determined using commercial standards. Quantification was performed using Sciex OS 1.6.1.29803 software. The samples were run in triplicate, as well as the pure compounds. The amount of phenolic components was calculated using calibration curves. Signal area of specific fragment ion was used for the quantitation (Table S1), and the results were expressed in µg/g dry weight of the plant (µg/g DW).

### 2.5. Antioxidant Activity of Veronica Extracts

#### 2.5.1. The Measurement of the ORAC Values

The assay was performed with a 96-well plate method, using black polystyrene microtiter plates (Porvair Sciences, Leatherhead, UK). Instrument for measurement of fluorescence was Tecan Infinite 200 PRO spectrophotometer (Tecan Trading AG, Männedorf, Switzerland). All experimental solutions and samples were prepared in a phosphate buffer (0.075 mM, pH 7.0, counter ion salts were NaH_2_PO_4_•H_2_O and Na_2_HPO_4_•7H_2_O.). Each reaction contained 180 µL of fluorescein (1 µM), 70 µL of 2,2′-Azobis(2-methyl-propionamidine) dihydrochloride (AAPH, Acros Organics) (300 mM) and 30 µL of blank (phosphate buffer), plant extract or reference standard Trolox (6.25–50 µM) (Sigma–Aldrich, St. Louis, MO, USA). The plate fills in this order: first column in all 8 wells, goes 30 µL of blank solution. Then in the next four columns, Trolox standard, each column different concentration, 6.25, 12.5, 25 and 50 µM (also 30 µL in each well). After Trolox, next columns are filled with samples, for each plant extract 2 columns with different concentrations (50 µg/mL and 25 µg/mL). After filling all wells with 30 µL of blank or Trolox or sample, in each well fluorescein is being added (amount mentioned above). The final step is adding AAPH in all wells, when the reaction and measurement on the instrument starts. Stock solutions of plant extracts were prepared in concentration of 1 mg/mL and then diluted in phosphate buffer to concentrations of 50 µg/mL and 25 µg/mL. At the end of ORAC experiment results were curves for blank, Trolox and samples. ORAC is calculated by measuring the Net Area Under the Curve (Net AUC) of a fluorescent probe’s decay in the presence and absence of an antioxidant, then comparing this to the decay of a Trolox standard and finally converting the results into Trolox equivalents (TEs) or micromole Trolox equivalents (µMTEs). First step is subtracting the blank AUC from the sample and standard AUCs then normalizing it against the Trolox standard and accounting for the dilution factor to express the final value in Trolox equivalents. The ORAC values of phenolic extracts were expressed as µmol of Trolox equivalents (TEs) per g of the dry phenolic extract. The results were obtained from three independent experiments.

#### 2.5.2. The Measurement of the DPPH Radical Scavenging Activity

The DPPH Radical Scavenging Activity was also analyzed with the 96-well plate method previously described by Mensor et al. and Payet et al. [18] and was adapted to the plant extracts tested. This method was also performed with a Tecan Infinite 200 PRO spectrophotometer (Tecan Trading AG, Switzerland) using 96-well transparent polystyrene microtiter plates (Porvair Sciences, Leatherhead, UK). Plant extracts were prepared in the concentration of 1 mg/mL (diluted from the primary stock solution of 10 mg of lyophilized extract dissolved in mL of distilled water). An amount of 100 µL of methanol (Kemika, Zagreb, Croatia) was pipetted in each well (except for the first column where blank was). Then 100 µL of the sample was pipetted into each well in the first row. Serial dilutions of the samples were prepared by pipetting 100 µL from the first row into the wells in the second row using a multichannel pipette and so on until the last row, where 100 µL of the solution was ejected after mixing. After this dilution, first row wells were mixed to a concentration of 500 µg/mL, 250 µg/mL and so on until the last one (eight row) with a concentration of 3.9 µg/mL. A blank sample was always added to the first column of the 96-well plates (phosphate buffer). Trolox was added to the second column, and samples were added to the other columns. The reaction starts with the addition of 100 µL of a DPPH (200 µM) methanolic solution to each well. The initial absorbance at 517 nm was measured immediately (it should be around 1.1 for the filled wells). After 30 min of incubation, the absorbance was measured again, and the percentage of DPPH inhibition was calculated according to the following formula from Yen and Duh [19]:

inhibition in % = ((AC(0) − AA(t))/AC(0)) × 100,

where AC(0) is the absorbance of the control at t = 0 min, and AA(t) is the absorbance of the antioxidant at t = 30 min. All measurements were performed in triplicate.

Because of the data from other relevant literature, we expressed results as IC50 values in µg of phenolic lyophilized compounds/mL of solution (lyophilized compounds dissolved in water).

### 2.6. Antibacterial Activity

Eight samples were tested for antibacterial activity: *V. anagallis-aquatica*, *V. hederifolia*, *V. persica* and *V. polita* from their natural habitat (NH) and from cultivation (C). Ethanol phenolic extracts were tested on eight bacterial species obtained from the ATCC (mainly Gram-positive since the previous tests have shown activity on Gram-positive bacteria). Methods of disk diffusion and diffusion performed by agar drilling have been used. At the same time, ATCC species of bacteria have been tested in accordance with EUCAST [20] so that the antibacterial activities can be compared (Tables S2–S9).

#### 2.6.1. Disk Diffusion

Empty paper sterile filter disks (6 mm × 1 mm) were used. Medium used for this testing, concentration of inoculum, incubation period, conditions and reading are defined in EUCAST Clinical Breakpoint Tables v. 15.0. This method is always the first choice for antibacterial susceptibility testing [21]. Tested bacterial material (ATCC bacterial strain) is usually marked as sensitive, moderately sensitive or resistant, and the method is considered as screening test for determination of antibacterial activity of herbal extracts, essential oils and other herbal preparations [22].

The disks for the diffusion were 6.3 mm × 1mm sterile paper disks that had capacity for maximum of 20 µL. On standardized nutrient medium, MH (Mueller–Hinton agar) and MH-F (Mueller–Hinton agar for Fastidious organisms) bacterial strains were inoculated (density 0.5 Mc Farland in sterile 0.9% NaCl solution) with very sensitive manual method that ensures coverage of the whole plate that is incubated for half an hour at 36 °C. After that, the disks impregnated with 20 µL were added [23,24]. The further procedure is the same as previously described in routine antibacterial testing—EUCAST Disk diffusion manual v 13.0 [25]. Density of inoculum was measured by Densimat densitometer (bioMerieux, Budapest, Hungary).

After 24 h of incubation we realized that there was no antibacterial activity which was related to the applied quantity (20 µL, 0.2 mg active substance), and therefore we raised the quantity to 100 µL (1mg active substance) because the tested solution was prepared in concentration of 10 mg/mL (the concentration of solution did not change just the volume used).

#### 2.6.2. Method of Diffusion by Agar Drilling

This method is very well known and commonly used, especially for herbal preparations [24,26]. Previously inoculated medium MH and MH-F is drilled with holes with 8 mm diameter with sterile plastic drillers. The holes (8 × 4 mm) are filled with 100 µL of phenol solution of examined *Veronica* species [26].

Extracts were tested on eight species of bacteria according to EUCAST recommendation and compared with two different quantities of extract from *Veronica* species (20 µL and 100 µL): *Escherichia coli* ATCC 25922, *Pseudomonas aeruginosa* ATCC 10145, *Listeria monocytogenes* ATCC 13932, *Listeria innocua* ATCC 33090, *Streptococcus pyogenes* ATCC 19615, *Staphylococcus aureus* ATCC 25923, *Enterococcus faecalis* ATCC 29212 and *Enterococcus faecium* ATCC 6057. Antibacterial susceptibility was tested using Mueller–Hinton agar with 5% defibrinated horse blood and 20 mg/L β NAD, inoculated with bacterial suspension 0.5 McFarland and incubated for 24 h at 37 °C according to EUCAST.

Empty sterilized antibiotic disks with diameter of 6 mm were used for testing antibacterial activity of *Veronica* phenol solution (Mastdiscs AST, Mast Group Ltd., UK).

### 2.7. Cytotoxic Analysis

The cytotoxic activity of phenolic compounds extracted from different *Veronica* species (*V. anagallis-aquatica*, *V. hederifolia*, *V. persica* and *V. polita*) from their natural habitat (NH) and from cultivation (C) was tested on cervical cancer cells (HeLa), colon cancer cells (HCT116), osteosarcoma cells (U2OS) and healthy human retinal pigment epithelium 1 (RPE1) cell lines (donated by prof. Janoš Terzić, School of Medicine, University of Split) using the MTS-based CellTiter 96^®^ Aqueous Assay (Promega, Madison, WI, USA), as described in Fredotović et al. [27]. After thawing, the cells were grown for 48 h at 37 °C in a humidified CO_2_ incubator. The relative humidity in the incubator was 95% with 5% CO_2_. When they reached the desired confluence (70–80% because the cells are in the exponential growth phase), they were counted with a Scepter 3.0 automatic handheld cell counter (Merck, Darmstadt, Germany) and seeded in 96-well plates containing serially diluted extracts from all *Veronica* species. Culture medium was DMEM (Dulbecco’s Modified Eagle Medium), High Glucose (4.5 g/L) with L-Glutamine (Capricorn Scientific GmbH, Ebsdorfergrund, Germany). The volume of pure medium per well was 50 µL, except for four wells in the last row, where it was 100 µL (blank). The number of cells seeded per well was 5000. The conditions were kept optimal for cell growth: 37 °C, 5% CO_2_ and 95% humidity. After a further 48 h, MTS tetrazolium reagent (Promega, Madison, WI, USA) was added to each well and left for a further 3 h, in the optimal conditions: 37 °C, 5% CO_2_ and 95% humidity. After this phase, absorbance was measured at 490 nm using a 96-well plate reader (Bio-Tek, EL808, Winooski, VT, USA). Each phenol concentration was tested in four replicates, and the IC_50_ value was calculated from three independent experiments.

### 2.8. Statistical Analysis

For the statistical analysis, a two-way ANOVA test was used for statistical processing of the data. To investigate the difference between the individual phenolic component present in the same species from natural habitats and from cultivation extracted with 80% ethanol and maceration extraction technique (different letters a–b), Sidak’s multiple comparison test was used. GraphPad Prism version 10 (GraphPad Software, Inc., San Diego, CA, USA) was used for statistical analysis.

## 3. Results

### 3.1. Phenolic Compounds

The aim of this work is to investigate the differences in the chemical profiles of the phenolic components of four species of the genus *Veronica* (*V. anagallis-aquatica*, *V. hederifolia*, *V. persica* and *V. polita*) from their natural habitat and from cultivation using 80% ethanol as a solvent and maceration as the extraction technique. The extraction yield was higher in the cultivated species for all samples except for *V. anagallis-aquatica* (Table 2). Out of the identified phenolics, the major phenolic compounds in all *Veronica* species (from their natural habitat and from cultivation) were as follows: apigenin, *p*-hydroxybenzoic acid, protocatechuic acid, gentisic acid, vanillic acid and caffeic acid (Table 3). The most abundant flavonoid in *Veronica* species was apigenin. From the natural habitat, the species *V. hederifolia* and *V. polita* have almost the same amount of apigenin (46.18 ± 5.12 µg/g DW and 48.53 ± 1.75 µg/g DW, respectively). It is interesting to note that quercetin and naringenin are compounds (flavonoids) that only occur in *Veronica* species from the natural habitat (Table 3). Rutin is present in almost all species of the genus *Veronica*, except in the cultivated *V. polita*. In general, it is characteristic of all four species of the genus *Veronica* that the cultivated species have fewer phenolic components than *Veronica* species from natural habitats (Table 3), and apigenin is present in smaller amounts in all cultivated species when compared to ones from the natural habitat.

### 3.2. Antioxidant Activity

The antioxidant activity was tested for wild and cultivated species in order to produce insights into the quality of the plant material that is cultivated in the gardens. The ethanol extract of the above-mentioned species was chosen as it was the extract that produced the best results in our previous research (along with the methanolic extract). The antioxidant activity was tested using two methods: ORAC (oxygen radical absorbance capacity) and DPPH (2,2-diphenyl-1-picrylhydrazyl).

#### 3.2.1. ORAC Activity

Table 4 shows the combined results from our previous research [17] and new results for cultivated species. The highest activity was recorded for cultivated *V. persica,* with a result of 3200 ± 190 µmol TE/g of DW. The second highest activity was determined for the wild *V. persica* extract, with an ORAC value of 3100 ± 270 µmol TE/g of DW, and cultivated *V. anagallis-aquatica*. Lowest activity was found for the wild *V. polita* extract, with 1300 ± 200 µmol TE/g of DW. In general, for all tested species, the cultivated species showed higher antioxidant activity.

#### 3.2.2. DPPH Activity

In the DPPH assay (Table 5), the highest activity was demonstrated for the extract of the cultivated *V. anagallis-aquatica,* with an IC50 value of 65 ± 8 µg/mL. The cultivated *V. persica* had a similar activity, with an IC50 value of 67 ± 5 µg/mL. The lowest activity was demonstrated for the wild *V. polita* extract, with an IC50 value of 474 ± 2 µg/mL. In general, similarly for the ORAC results, all extracts of cultivated species showed better antioxidant activity than the wild ones.

### 3.3. Antibacterial Activity

**Figure 1:**
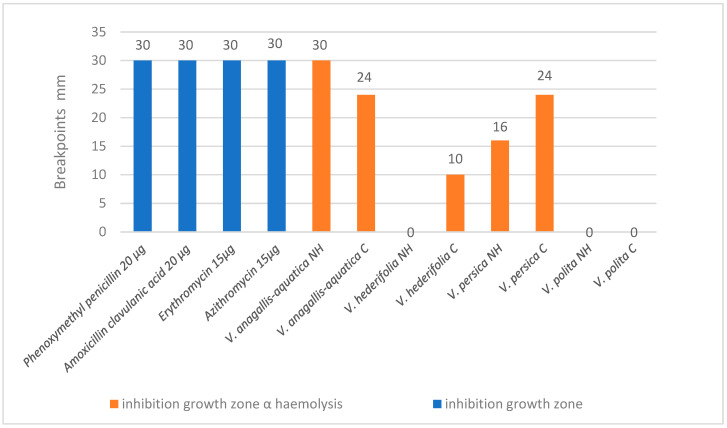
Results for antibacterial susceptibility of *S. pyogenes* ATCC 19615 (partially because the inhibition zones are visible as α haemolysis).

For the antibacterial activity, results for the bacteria strains on which tested extracts showed inhibition are presented. The chosen *Veronica* extracts had an “antibacterial response” to the three tested bacteria, all Gram-positive bacteria. First is *Streptococcus pyogenes* ATCC 19615 (half-heartedly because the inhibition zones are visible as α haemolysis), and the difference between two zones (α and β) is clearly visible (Figure 1). The *Veronica* species that demonstrated a substantial inhibition comparable to the antibiotic were as follows: cultivated and natural *V. anagallis-aquatica* and cultivated *V. persica*. The differences between the results for the natural and cultivated species were statistically significant for all species except for *V. polita*. If the concentration of the active substance was increased, maybe the results would be comparable with zones of the growth inhibition produced with tested antibiotic disks. This especially needs to be further investigated for the phenolic solutions of wild *V. anagallis-aquatica* and cultivated *V. persica*.

**Figure 2:**
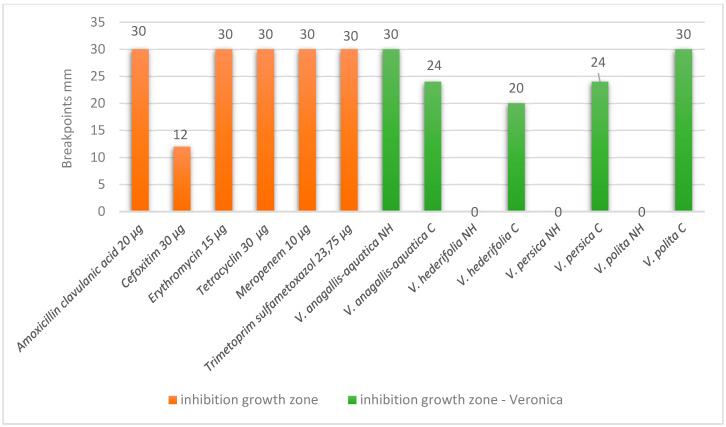
Results for antibacterial susceptibility of *L. monocytogenes* ATCC 13932.

The second bacteria that our extracts showed antibacterial activity is *Listeria monocytogenes* ATCC 13932 (Figure 2). The antibacteriological testing shows very good results, especially for the wild and cultivated *V. anagallis-aquatica* and for the cultivated *V. polita*. The differences in the results between the NH and cultivated species were statistically significant for all species.

**Figure 3:**
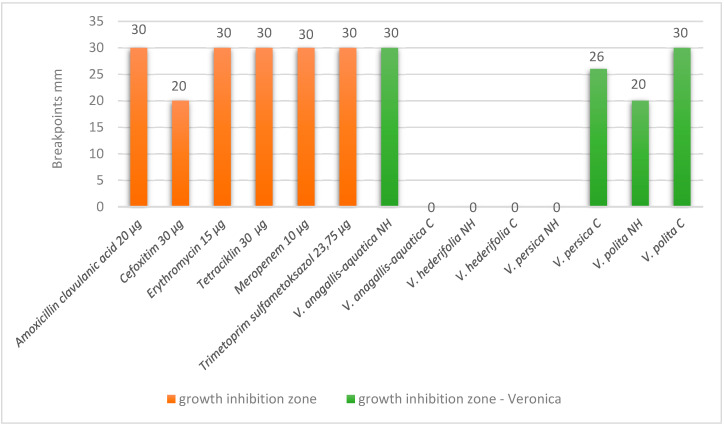
Results for antibacterial susceptibility of *L. innocua* ATCC 33090.

The third bacteria for which our extracts showed antibacterial activity is *Listeria innocua* ATCC 33090 (Figure 3). The antibacteriological testing shows very good results, especially for *V. anagallis-aquatica* from the natural habitat, for cultivated *V. persica* and for cultivated *V. polita*. The differences between the results for the natural habitat and cultivated species were statistically significant for all species except for *V. hederifolia*.

### 3.4. Cytotoxic Activity

**Figure 4:**
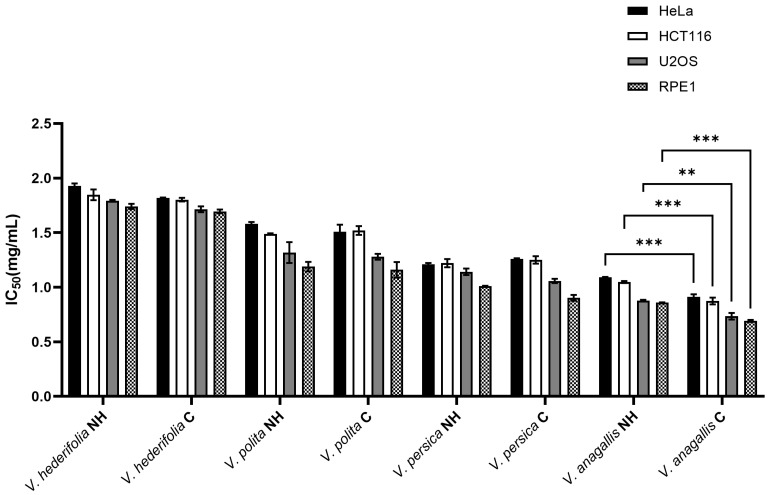
Cytotoxic activity of phenolic compounds extracted from *V. anagallis-aquatica*, *V. hederifolia*, *V. persica* and *V. polita* from their natural habitat (NH) and from cultivation (C) on the cervical (HeLa), colon (HCT116) and osteosarcoma (U2OS) cell line and on the healthy human retinal pigment epithelium 1 (RPE1) cell line using the MTS cell-based proliferation assay. The IC_50_ values are mean values from three individual experiments with standard deviation. For statistical analysis, a two-way ANOVA was performed followed by Turkey’s multiple comparison test. A statistically significant difference between NH and C plants is indicated by ** for *p* < 0.01 and *** for *p* < 0.001.

The cytotoxic activity of phenolic compounds extracted from various *Veronica* species (*V. anagallis-aquatica*, *V. hederifolia*, *V. persica* and *V. polita*) from their natural habitat (NH) and from cultivation (C) were tested on three cell lines, HeLa, HCT116 and U2OS, as well as on a healthy human retinal pigment epithelium 1 (RPE1) cell line (Figure 4). The results showed differences between the species and the cultivation method. The phenolic compounds isolated from the *V. anagallis-aquatica* species showed the best cytotoxic effect on all tested lines, while the phenolic compounds from the species that were cultured showed a slightly better effect. The IC_50_ values ranged between 0.77 mg/mL (C) and 0.86 mg/mL (NH) for the RPE1 cell line, 0.78 mg/mL (C) and 0.88 mg/mL (NH) for U2OS, 0.87 mg/mL (C) and 1.05 mg/mL (NH) for HCT116 and 0.91 mg/mL (C) and 1.09 mg/mL (NH) for the HeLa cell line. The chemical analysis of the phenolics of *V. anagallis-aquatica* revealed the highest levels of *p*-hydroxybenzoic acid, chlorogenic acid and apigenin compared to the other species tested, suggesting that they may be responsible for significant cytotoxic activity. Besides *V. anagallis-aquatica*, the phenolic compounds isolated from *V. persica* also showed significant results of cytotoxic activity, while the phenolics isolated from *V. polita* showed slightly weaker activity, and the phenolics from *V. hederifolia* showed the least activity. The *p*-hydroxybenzoic acid, chlorogenic acid and apigenin were also the dominant compounds isolated from *V. persica*, similar to *V. anagallis-aquatica*, but interestingly, rutin was the most abundant in the cultured *V. persica* of all species studied, which probably plays a role in the cytotoxic potential of this species. The phenolics isolated from *V. persica* from the natural habitat (NH) and from the culture showed almost no differences in the ability to stop the cell division of all cell lines tested. Interestingly, the phenolic compounds from almost all *Veronica* samples showed comparable cytotoxic activity on the healthy cell line RPE1, with IC_50_ values between 0.78 mg/mL and 1.69 mg/mL, and the U2OS carcinoma cell line (IC_50_ values between 0.87 mg/mL and 1.89 mg/mL).

## 4. Discussion

The aim of this research is to analyze the chemical profiles of different *Veronica* species from natural habitats and from cultivation using 80% ethanol as a solvent and maceration as the extraction technique. Conventional extraction techniques, such as maceration, have advantages, such as the simplicity of their implementation and low operating costs, but also disadvantages, such as long extraction times, large amounts of solvent and lower yields [28,29]. Vrca et al. 2025 [29] reported a better extraction yield using an ultrasound-assisted extraction (UAE) technique than using the maceration technique, thanks to ultrasonic waves that participate in breaking or deforming the cell wall, which enhances the extraction of desired bioactive components and thus leads to better yields compared to traditional techniques [30,31]. This study did not examine a comparison of different extraction techniques but rather used maceration as the most common extraction technique. Vrca et al. 2024 reported that the main compounds present in *V. anagallis-aquatica*, *V. persica* and *V. polita* were *p*-hydroxybenzoic acid, vanillic acid, caffeic acid, gentisic acid and apigenin [17]. In this research, results for new species (*V. hederifolia*), for cultivated versions of three mentioned species from the previous research by Vrca et al. [17] and for a new one (*V. hederifolia*) were investigated. Generally, the highest amount of phenolic components was in *V. anagallis-aquatica*, which is the result of our previous work [17]. Just looking at the amounts of the phenolic compound in the dry material mass, it can be concluded that cultivated species have lower quantities of extracted phenolics, which is logical as the species that are cultivated mostly live in the conditions that are not stressful at any time. According to Barreira et al. (2014), *V. montana* and *V. spuria* had a similar amount of phenolic acids, while *V. polita* had the lowest [7]. Our obtained results show that *V. hederifolia* and also *V. polita* had the lowest content of phenolic components compared to the other two species: *V. anagallis aquatica* and *V. persica*. Also, the highest amount of phenolic compounds was found in *V. montana* (Barreira et al., 2014) [7]. According to Beara et al., (2015), Kwak et al., (2009), Stojković et al., (2013) and Albach et al., (2003) *Veronica* species contain various phenolic compounds, most of which are considered beneficial to human health, which is also in accordance with our results [3,32,33,34]. Cohen & Kennedy (2010) reported that the difference in the chemical profile of phenols can be attributed to the availability of water, exogenous growth and development factors, the availability of light and various parasites [35]. The above reasons can justify our results that show that plants of the genus *Veronica* from natural habitats have a higher content of phenolic components compared to cultivated species, precisely because of biotic and abiotic factors.

*Veronica* species have been known for their use in the treatment of various diseases, including various forms of cancer [36,37,38]. In the past, little research has been performed on their possible biological effects. However, as the species of this genus are rich in biologically active compounds, there is a need for more detailed studies of their activities, especially their antimicrobial, antioxidant, anti-inflammatory, cytotoxic and anticarcinogenic effects. So far, mainly methanolic, ethanolic and aqueous extracts have been tested, as well as essential oils and hydrosols as by-products of essential oils in more recent studies [1,39,40,41,42]. Studies by Vrca et al. on essential oils and hydrosols of six *Veronica* species (*V. agrestis*, *V. anagalloides*, *V. austriaca* ssp. *jacquinii*, *V. beccabunga*, *V. cymbalaria* and *V. officinalis*) have interestingly shown that hydrosols of all tested species have a stronger antiproliferative effect on the MDA-MD-231 and T24 cell lines than essential oils. The essential oil of *V. agrestis* showed the greatest ability to induce apoptosis in the MDA-MD-231 cell lines of all tested oils, while *V. cymbalaria* and *V. anagalloides* had the best apoptotic effect among the tested hydrosols [43]. More studies were carried out with methanol extracts from various *Veronica* species. Harput et al. demonstrated that chloroform fractions obtained by the fractionation of methanol extracts from five *Veronica* species (*V. cymbalaria*, *V. hederifolia*, *V. pectinata* var. *glandulosa*, *V. persica* and *V. polita*) have a dose-dependent cytotoxic effect on two carcinoma cell lines, KB (human epidermoid carcinoma) and B16 (mouse melanoma) [44]. In another study, methanolic extracts of eight species from four families of Lamiales, *Limosella aquatica* L. (Scrophulariaceae), *Mimulus glabratus* Kunth (Phrymaceae), *Pedicularis mexicana* Zucc. ex Benth. (Orobanchaceae), *Penstemon campanulatus* (Cav.) Willd. (Plantaginaceae) and *Veronica americana* (Raf.) Schwein (Plantaginaceae), showed that of all the species tested, *V. americana* had the highest cytotoxic activity against PC-3 (prostate cancer) and HF-6 (colon cancer) cells, with IC_50_ values of 1.46 and 0.169 μg/mL, respectively [45]. Flavonoids isolated from *V. sibirica* (Vtfs) inhibited growth and induced apoptosis in breast cancer cells (MCF-7) with a significantly low IC_50_ concentration of 42 ug/mL [46]. The studies mentioned above speak of a significant anti-cancer potential of various extracts of *Veronica* species. The present study is one of the few in which phenolic compounds isolated from species of the genus *Veronica* from their natural habitat and from cultivation were tested on cancer cell lines (HeLa, HCT116 and U2OS) and a healthy cell line (RPE1). The results showed differences in the cytotoxic potential between species and cultivation methods, which certainly indicates the need for further research to find the species with the highest content of bioactive phenolic compounds and possible chemotherapeutic properties.

In general, the results for antioxidant activity show that extracts from cultivated species have higher activity in both methods (ORAC and DPPH). If we look at the results for the chemical composition of these extracts in comparison to extracts from the natural habitat species, apigenin is present in all plant extracts in the highest concentration when comparing it to other identified compounds. But apigenin is present in smaller amounts in all cultivated species in comparison to the natural habitat ones. Andueza et al. investigated pro-oxidant activity of apigenin [47], and they found that apigenin can cause faster oxidation of some fluorescent probes, so this could be the reason why extracts of cultivated species have higher antioxidant activity. If species are compared, the highest antioxidant activities in both methods come from extracts from the cultivated *V. persica* and *V. anagallis-aquatica*. *V. persica* has the smallest amount of apigenin, so this could be the reason for the high antioxidant activity, and it is the only extract that contains quinic acid, which is known for its antioxidant activity [48]. This possibility of apigenin as being responsible for lower antioxidant activity should be further investigated. There are many other reported results for the antioxidant activity of phenolic compounds of *Veronica* species. For example, Živković et al. reported higher DPPH antioxidant activity for *V. teucrium* and *V. jacquinii*, 28.49 ± 0.6 µg/mL and 37.63 ± 0.6 µg/mL, respectively. Harput et al. reported similar DPPH antioxidant activity for methanolic extracts of *V. peduncularis*, with an IC50 value of 54.19 µg/mL. In a study by Nikolova M., DPPH activity was tested for *V. urticifolia* and *V. serpyllifolia*, and it was higher than 200 µg/mL, but for other *Veronica* species like *V. officinalis*, *V. vindobonensis* and *V. chamaedrys* a higher DPPH activity was reported that is comparable to other medicinal plants, such as *Clinopodium vulgarae*, *Clematis vitalba* and *Stachys recta* [49]. Ninfali et al. reported ORAC values for some common medicinal herbs. Results from our study are lower than those of some common herbs, such as *Salvia officinalis*, but are similar to the activity of *Rosmarinus officinalis* [50]. Zheng et al. reported the ORAC value for Mexican oregano (*Poliomintha longiflora*). The ORAC value for *V. polita* methanolic extract was similar to the one reported for Mexican oregano, 5121 µg/g phenolic compounds [51]. Wojcikowski et al. reported ORAC values for 55 medicinal herbs used for treating the urinary system, and according to these values, the ORAC activity of all three species tested in our study is higher than the herbs reported in the mentioned work [52]. In the recent research on *V. officinalis*, the DPPH activity IC50 value was 45 µg/mL, which is slightly higher than the highest activity from our research for *V. anagallis-aquatica* and *V. persica* [53].

In general, herbs can be used within the form of plant extracts or as their active components. They can be used either in combination with traditional medication or alone within appropriate preparation [54]. The extracts of herbal materials signify continuous attempts to investigate new compounds with potential antibacterial activity. Antibiotics and other chemical antimicrobial agents play a big role as antimicrobial agents, but they lead to various side effects. In relations to this, bioavailability is the primary essential measure for assessing the health benefits of herbal phenolic compounds. The antibacterial activity of the investigated extracts was good against two *Listeria* species. There are several species in the genus *Listeria*. Of these, *L. monocytogenes* is important as a cause of a wide spectrum of diseases among animals and humans [55]. Of all the investigated species from this study, both the cultivated and natural *V. anagallis-aquatica* showed very good antibacterial activity against *L. monocytogenes* and *Streptococcus pyogenes*. These extracts have higher amounts of quercetin, rutin and caffeic acid, which have shown good antibacterial activities when investigated as standards [56,57]. The cultivated *V. persica* showed good results against *L. monocytogenes*, *L. innocua* and *S. pyogenes*. This extract is the only one that has quinic acid, so this could be the reason for this good activity [58]. *Veronica polita* from natural habitat and cultivated groups was very good against *L. innocua*. These results could be due to the presence of *p*-coumaric acid, which has showed very good activity against various bacterial strains in research by Lou et al. [59]. If compared to other research of antibacterial activity by disk diffusion tests, our results for the above-mentioned extracts stand very high above some recent studies. Elagdi et al. investigated methanolic extracts of *Euphorbia resinifera* and *Euphorbia echinus* and had a restriction area of 7–12 mm [60]. Darabpour et al. investigated different concentrations of *Teucrium polium* extract, and the highest result of a 26 mm inhibition diameter was reported for the methanolic extract concentration of 600 mg/mL against *Bordetella bronchiseptica* [61]. In the recent research on *V. officinalis*, Horozić et al. reported smaller inhibition zones than those for the species from our research, but they used lower concentrations for the extracts (50–100 μg/mL). Dulger et al. reported antibacterial activity for various plant species, and the inhibition zones for all species were lower than the results from our research of the three most active species. Also, the difference in the amount of the active substance is notable [62].

## 5. Conclusions

In this study, four species were investigated: *V. anagallis-aquatica*, *V. persica*, *V. polita* and *V. hederifolia*. Ethanol phenolic extracts were analyzed for cultivated and natural habitat species. Also, the antioxidant, antibacterial and cytotoxic activity was tested for each extract. In general, the most abundant compound for all species was apigenin, but it had higher amounts in the natural habitat species. *V. anagallis-aquatica* and *V. persica* showed the best results for all biological activities tested. For these species, cultivated ones showed somewhat better results. These are very promising results in terms of using these extracts further. The fact that cultivated species have abundant biologically active substances presents a better possibility of using these plants commercially without endangering their natural habitats. As a result of this study, it can be concluded that natural habitat and cultivated species have different phenolic chemical compositions and thus biological activities. Further studies should include investigating phenolic extracts from other *Veronica* species, to finally conclude which species is most valuable in the terms of biological activity that could be further applied in possible medical supplements. Also, further research could include more concentrated extract solutions, which could show even better results for antibacterial activity. This kind of information is crucial, especially in this antibiotic resistance era, and provides wider therapeutic possibilities.

## Figures and Tables

**Table 1:** Localities of the wild *Veronica* species.

Taxa	Locality	Latitude	Longitude	Altitude a.s.l. (m)	Voucher No.
*V. anagallis-aquatica* L. [17] *	Split	43°31′44.7″ N	16°28′45.8″ E	22	CROVeS-01-2022
*V. persica* Poir. [17] *	Hvar	43°09′42.8″ N	16°40′37.6″ E	18	CROVeS-22-2022
*V. polita* Fr. [17] *	Hvar	43°10′42.3″ N	16°36′43.6″ E	38	CROVeS-23-2022
*V. hederifolia* L.	Zaton Obrovački	44°13′00.1″ N	15°40′49.91″ E	135	CROVeS-24-2022

* Ethanolic extract of these three species from natural habitat were previously reported in Vrca et al. 2024 [17].

**Table 2:** Comparison of extraction yield of four different *Veronica* species from their natural habitat (NH) and cultivated habitat (C).

Extracts	*V. anagallis-aquatica*		*V. hederifolia*		*V. persica*		*V. polita*	
Extraction yield (%)								
80%-ethanol	NH	C	NH	C	NH	C	NH	C
	32 [17]	27	32	34	18 [17]	24	14 [17]	19

Note: Extraction yield was calculated as µg of liophilized phenolic compounds/g of dry plant material.

**Table 3:** Identification and quantification of the phenolic components in ethanolic extracts of four *Veronica* species (*V. anagallis-aquatica*, *V. hederifolia*, *V. persica* and *V. polita*) from natural habitats (NHs) and from cultivation (C) (µg/g DW).

	*V. anagallis-aquatica*		*V. hederifolia*		*V. persica*		*V. polita*	
Compound	80% EtOH Extract (NH) *	80% EtOH Extract (C)	80% EtOH Extract (NH)	80% EtOH Extract (C)	80% EtOH Extract (NH) *	80% EtOH Extract (C)	80% EtOH Extract (NH) *	80% EtOH Extract (C)
*p*-hydroxybenzoic acid	5.0 ± 0.36	2.3 ± 0.25	0.14 ± 0.010	0.05 ± 0.000	2.9 ± 0.16	0.48 ± 0.020	0.54 ± 0.020	0.13 ± 0.010
Protocatechuic acid	3.9 ± 0.13	2.1 ± 0.29	0.36 ± 0.010	0.12 ± 0.010	4.8 ± 0.37	2.97 ± 0.43	0.24 ± 0.004	0.11 ± 0.010
Gentisic acid	3.9 ± 0.20	2.0 ± 0.31	0.36 ± 0.030	0.13 ± 0.010	4.9 ± 0.24	2.86 ± 0.38	0.27 ± 0.010	0.09 ± 0.000
Vanillic acid	3.0 ± 0.18	0.62 ± 0.080	1.31 ± 0.05	0.41 ± 0.050	2 ± 0.2	0.46 ± 0.030	0.36 ± 0.040	0.15 ± 0.010
Gallic acid	0.38 ± 0.010	0.19 ± 0.000	n.d.	n.d.	1.1 ± 0.08	0.17 ± 0.000	0.18 ± 0.002	n.d.
Syringic acid	n.d.	n.d.	n.d.	n.d.	n.d.	n.d.	n.d.	n.d.
*p*-coumaric acid	1.3 ± 0.01	0.24 ± 0.020	0.06 ± 0.000	0.04 ± 0.000	0.64 ± 0.020	0.13 ± 0.000	1.7 ± 0.08 ^a^	0.10 ± 0.000 ^b^
*o*-coumaric acid	n.d.	n.d.	n.d.	n.d.	n.d.	n.d.	0.09 ± 0.001	n.d.
Caffeic acid	7.5 ± 0.06	0.69 ± 0.120	0.44 ± 0.020	0.11 ± 0.010	3.9 ± 0.04	1.84 ± 0.28	1.2 ± 0.04 ^a^	0.13 ± 0.010 ^b^
Ferulic acid	0.54 ± 0.020	0.14 ± 0.000	0.08 ± 0.000	0.02 ± 0.000	0.30 ± 0.010	0.19 ± 0.01	0.14. ± 0.000	0.10 ± 0.000
Chlorogenic acid	3.0 ± 0.01	1.3 ± 0.20	0.11 ± 0.000 ^a^	0.08 ± 0.000 ^a^	0.26 ± 0.010	4.62 ± 1.42	0.40 ± 0.010	0.13 ± 0.000
Quinic acid	n.d.	n.d.	n.d.	n.d.	n.d.	3.44 ± 0.55	n.d.	n.d.
Sinapic acid	n.d.	n.d.	n.d.	n.d.	n.d.	n.d.	n.d.	n.d.
Rosmarinic acid	n.d.	n.d.	n.d.	n.d.	n.d.	n.d.	n.d.	n.d.
Cinnamic acid	n.d.	n.d.	n.d.	n.d.	n.d.	n.d.	n.d.	n.d.
Epicatechin	n.d.	n.d.	n.d.	n.d.	n.d.	n.d.	n.d.	n.d.
Catechin	n.d.	n.d.	0.36 ± 0.000	n.d.	n.d.	n.d.	n.d.	n.d.
Resveratrol	n.d.	n.d.	n.d.	n.d.	n.d.	n.d.	n.d.	n.d.
Astringin	n.d.	n.d.	n.d.	n.d.	n.d.	n.d.	n.d.	n.d.
EGCG (Epigallocatechin gallate)	n.d.	n.d.	n.d.	n.d.	n.d.	n.d.	n.d.	n.d.
Hesperetin	n.d.	n.d.	n.d.	n.d.	n.d.	n.d.	n.d.	n.d.
Quercetin	3.2 ± 0.22	n.d.	0.52 ± 0.000	n.d.	2.2 ± 0.13	n.d.	1.2 ± 0.02	n.d.
Myricetin	n.d.	n.d.	n.d.	n.d.	n.d.	n.d.	n.d.	n.d.
Apigenin	950 ± 22 ^a^	113 ± 5 ^b^	46 ± 5.1	10 ± 3.1	661 ± 26 ^a^	6.4 ± 0.51 ^b^	48 ± 1.8 ^a^	0.81 ± 0.060 ^b^
Naringenin	0.64 ± 0.030	n.d.	0.08 ± 0.000	n.d.	0.56 ± 0.020	n.d.	0.22 ± 0.010	n.d.
Rutin	3.5 ± 0.17	1.8 ± 0.05	2.4 ± 0.32	0.26 ± 0.010	1.0 ± 0.07	5.1 ± 1.10	0.59 ± 0.030	n.d.

Legend: n.d.—not detected; dw—dry weight. Data are presented as mean ± SD (standard deviation), n = 3. A two-way ANOVA test was used for statistical data processing. Then, Sidak’s multiple comparison test was used to investigate the difference between the individual components present in the same species (wild and cultivated) extracted with 80% ethanol and maceration extraction technique (different letters a–b): *—results from our previous work, Vrca et al. [17].

**Table 4:** Antioxidant activity of four wild and cultivated *Veronica* species in ORAC method.

Species	Natural Habitat (NH)	Cultivated (C)
*V. hederifolia*	1700 ± 100 ^aB^	2000 ± 35 ^aB^
*V. polita*	1300 ± 200 *^bB^	3000 ± 100 ^aA^
*V. persica*	3100 ± 270 *^aA^	3200 ± 190 ^aA^
*V. anagallis-aquatica*	2700 ± 370 *^aA^	3100 ± 120 ^aA^

ORAC, oxygen radical absorbance capacity, results for phenolic extracts expressed as µmol of Trolox equivalents (TEs) per g of DW of extracted phenolic compounds; *—results from our previous work, Vrca et al. [17]; a two-way ANOVA test was used for statistical data processing, denoted with capital letters A–B in the columns, comparing antioxidant activity between different species, after which Tukey’s multiple comparison test was used to examine the difference between antioxidant activity in the same natural habitat/cultivated species, denoted with letters a–b in the rows; activities with the same letters in the rows (a–b) mean that there is no statistically significant difference between wild and cultivated of the same species; and activities with the same letters in the columns (A–B) mean that there is no statistically significant difference between different species.

**Table 5:** Antioxidant activity of four wild and cultivated *Veronica* species in DPPH method.

Species	Natural Habitat (NH)	Cultivated (C)
*V. hederifolia*	341 ± 25 ^bB^	240 ± 20 ^aB^
*V. polita*	474 ± 2 *^bC^	72 ± 3 ^aA^
*V. persica*	121 ± 11 *^bA^	67 ± 5 ^aA^
*V. anagallis-aquatica*	127 ± 17 *^bA^	65 ± 8 ^aA^

DPPH, results for phenolic extracts expressed as IC50 in µg/m; SD = standard deviation of triplicate analysis; and *—results from our previous work, Vrca et al. [17]. A two-way ANOVA test was used for statistical data processing, denoted with capital letters A–C in the columns, comparing antioxidant activity between different species, after which Tukey’s multiple comparison test was used to examine the difference between antioxidant activity in the same wild/cultivated species, denoted with letters a–b in the rows; activities with the same letters in the rows (a–b) mean that there is no statistical significance; and activities with the same letters in the columns (A–C) mean that there is no statistical significance.

## Data Availability

The samples and any additional research data are available from the authors on request.

## Supplementary Materials

The following supporting information can be downloaded at https://www.mdpi.com/article/10.3390/antiox14111308/s1: Table S1. Precursor and quantitation fragments *m*/*z* and retention time of phenolic compounds; Tables S2–S9: Antibacterial testing of antibiotics according to EUCAST.

## References

[1] Salehi B., Shetty M.S., Anil Kumar N.V., Živković J., Calina D., Docea A.O., Emamzadeh-Yazdi S., Kılıç C.S., Goloshvili T., Nicola S. (2019). Veronica Plants—Drifting from Farm to Traditional Healing, Food Application, and Phytopharmacology. Molecules.

[2] Mocan A., Vodnar D.C., Vlase L., Crișan O., Gheldiu A.M., Crișan G. (2015). Phytochemical Characterization of *Veronica officinalis* L., V. *Teucrium* L. and V. *Orchidea crantz* from Romania and Their Antioxidant and Antimicrobial Properties. Int. J. Mol. Sci..

[3] Albach D.C., Grayer R.J., Jensen S.R., Özgökce F., Veitch N.C. (2003). Acylated Flavone Glycosides from Veronica. Phytochemistry.

[4] Xue H., Chen K.X., Zhang L.Q., Li Y.M. (2019). Review of the Ethnopharmacology, Phytochemistry, and Pharmacology of the Genus Veronica. Am. J. Chin. Med..

[5] Taskova R.M., Kokubun T., Ryan K.G., Garnock-Jones P.J., Jensen S.R. (2010). Phenylethanoid and Iridoid Glycosides in the New Zealand Snow Hebes (Veronica, Plantaginaceae). Chem. Pharm. Bull..

[6] Marchenko A., Kintya P., Wyrzykiewicz B., Gorincioi E. (2012). Steroidal Glycosides from *Veronica chamaedrys* L. Part I. The Structures of Chamaedrosides C, C1, C2, E, E1and E 2. Nat. Prod. Commun..

[7] Barreira J.C.M., Dias M.I., Živković J., Stojkovic D., Soković M., Santos-Buelga C., Ferreira I.C.F.R. (2014). Phenolic Profiling of *Veronica* Spp. Grown in Mountain, Urban and Sandy Soil Environments. Food Chem..

[8] Dai J., Mumper R.J. (2010). Plant Phenolics: Extraction, Analysis and Their Antioxidant and Anticancer Properties. Molecules.

[9] Santos-Buelga C., Escribano-Bailon M.T., Lattanzio V. (2010). International Conference on Polyphenols (24th: 2008: Salamanca, S). Recent Advances in Polyphenol Research.

[10] Živković J., Ćebović T., Maksimović Z. (2012). In Vivo and in Vitro Antioxidant Effects of Three Veronica Species. Cent. Eur. J. Biol..

[11] Živković J., Barreira J.C.M., Stojković D., Ćebović T., Santos-Buelga C., Maksimović Z., Ferreira I.C.F.R. (2014). Phenolic Profile, Antibacterial, Antimutagenic and Antitumour Evaluation of *Veronica urticifolia* Jacq. J. Funct. Foods.

[12] Vitaglione P., Morisco F., Caporaso N., Fogliano V. (2004). Dietary Antioxidant Compounds and Liver Health. Crit. Rev. Food Sci. Nutr..

[13] Pokorný J. (2007). Are Natural Antioxidants Better—And Safer—Than Synthetic Antioxidants?. Eur. J. Lipid Sci. Technol..

[14] Lobiuc A., Pavăl N.E., Mangalagiu I.I., Gheorghiță R., Teliban G.C., Amăriucăi-Mantu D., Stoleru V. (2023). Future Antimicrobials: Natural and Functionalized Phenolics. Molecules.

[15] Parham S., Kharazi A.Z., Bakhsheshi-Rad H.R., Nur H., Ismail A.F., Sharif S., Ramakrishna S., Berto F. (2020). Antioxidant, Antimicrobial and Antiviral Properties of Herbal Materials. Antioxidants.

[16] Muluneh M.G. (2021). Impact of Climate Change on Biodiversity and Food Security: A Global Perspective—A Review Article. Agric Food Secur.

[17] Vrca I., Orhanović S., Pezelj I., Sušić K., Dunkić V., Kremer D., Nazlić M. (2024). Identification of Phenolic Compounds Present in Three Speedwell (*Veronica* L.) Species and Their Antioxidant Potential. Antioxidants.

[18] Mensor L.L., Menezes F.S., Leitão G.G., Reyes A.S., dos Santos T.C., Fit C.S., Leitão S.G. (2001). Screening of Brazilian Plant Extracts for Antioxidant Activity by the Use of DPPH Free Radical Method. Phytother. Res..

[19] Yen G.-C., Duh P.-D. (1994). Scavenging Effect of Methanolic Extracts of Peanut Hulls on Free-Radical and Active-Oxygen Species. J. Agric. Food Chem..

[20] Matuschek E., Brown D.F.J., Kahlmeter G. (2014). Development of the EUCAST Disk Diffusion Antimicrobial Susceptibility Testing Method and Its Implementation in Routine Microbiology Laboratories. Clin. Microbiol. Infect..

[21] Koneman E.W., Winn W.C., Allen S.D., Janda W.M., Schreckenberger P.C., Procop G.W., Woods G.L. (2006). Koneman’s Color Atlas and Textbook of Diagnostic Microbiology.

[22] Nazlić M., Dunkić V., Dželalija M., Maravić A., Mandić M., Srečec S., Vrca I., Vuko E., Kremer D. (2023). Evaluation of Antiphytoviral and Antibacterial Activity of Essential Oil and Hydrosol Extracts from Five *Veronica* Species. Agriculture.

[23] Çelik E., Yuvali G., Meysun I.A. (2010). Essential Oil Composition and Antibacterial Activity of Some Plant Species. J. Appl. Biol. Sci..

[24] Hemeg H.A., Moussa I.M., Ibrahim S., Dawoud T.M., Alhaji J.H., Mubarak A.S., Kabli S.A., Alsubki R.A., Tawfik A.M., Marouf S.A. (2020). Antimicrobial Effect of Different Herbal Plant Extracts against Different Microbial Population. Saudi J. Biol. Sci..

[25] (2025). EUCAST Disk Diffusion Method for Antimicrobial Susceptibility Testing Antimicrobial Susceptibility Testing EUCAST Disk Diffusion Method. https://www.eucast.org/fileadmin/src/media/PDFs/EUCAST_files/Disk_test_documents/2025_manuals/Manual_v_13.0_EUCAST_Disk_Test_2025.pdf.

[26] Cowan M.M. (1999). Plant Products as Antimicrobial Agents. Clin Microbiol Rev..

[27] Fredotović Ž., Soldo B., Šprung M., Marijanović Z., Jerković I., Puizina J. (2020). Comparison of Organosulfur and Amino Acid Composition between Triploid Onion Allium Cornutum Clementi Ex Visiani, 1842, and Common Onion *Allium cepa* L., and Evidences for Antiproliferative Activity of Their Extracts. Plants.

[28] Mathews A., Arbal A.V., Kaarunya A., Jha P.K., Le-Bail A., Rawson A. (2024). Conventional vs Modern Extraction Techniques in the Food Industry. Extr. Process. Food Ind..

[29] Vrca I., Jukić D., Radić J., Anđelić I. (2025). Phenolic Compounds in Edible *Tropaeolum majus* L. Leaves and Its In Vitro Digestion. Analytica.

[30] Shen L., Pang S., Zhong M., Sun Y., Qayum A., Liu Y., Rashid A., Xu B., Liang Q., Ma H. (2023). A Comprehensive Review of Ultrasonic Assisted Extraction (UAE) for Bioactive Components: Principles, Advantages, Equipment, and Combined Technologies. Ultrason. Sonochem.

[31] Mnayer D., Fabiano-Tixier A.-S., Petitcolas E., Ruiz K., Hamieh T., Chemat F. (2017). Extraction of Green Absolute from Thyme Using Ultrasound and Sunflower Oil. Resour.-Effic. Technol..

[32] Beara I., Živković J., Lesjak M., Ristić J., Šavikin K., Maksimović Z., Janković T. (2015). Phenolic Profile and Anti-Inflammatory Activity of Three Veronica Species. Ind. Crops Prod..

[33] Kwak J.H., Kim H.J., Lee K.H., Kang S.C., Zee O.P. (2009). Antioxidative Iridoid Glycosides and Phenolic Compounds from Veronica Peregrina. Arch. Pharm. Res..

[34] Stojković D.S., Živković J., Soković M., Glamočlija J., Ferreira I.C.F.R., Janković T., Maksimović Z. (2013). Antibacterial Activity of *Veronica montana* L. Extract and of Protocatechuic Acid Incorporated in a Food System. Food Chem. Toxicol..

[35] Cohen S.D., Kennedy J.A. (2010). Plant Metabolism and the Environment: Implications for Managing Phenolics. Crit. Rev. Food Sci. Nutr..

[36] Graham J.G., Quinn M.L., Fabricant D.S., Farnsworth N.R. (2000). Plants Used against Cancer—An Extension of the Work of Jonathan Hartwell. J. Ethnopharmacol..

[37] Su B.N., Zhu Q.X., Jia Z.J. (1999). Aquaticol, a Novel Bis-Sesquiterpene from Veronica Anagallis-Aquatica. Tetrahedron Lett..

[38] Tomassini L., Brkic D., Serafini M., Nicoletti M. (1995). Constituents of Veronica Hederifolia and Veronica Polita. Fitoterapia.

[39] Nazlić M., Fredotović Ž., Vuko E., Fabijanić L., Kremer D., Stabentheiner E., Ruščić M., Dunkić V. (2021). Wild Species *Veronica officinalis* L. and *Veronica saturejoides* Vis. Ssp. Saturejoides—Biological Potential of Free Volatiles. Horticulturae.

[40] Nazlić M., Fredotović Ž., Vuko E., Vuletić N., Ljubenkov I., Kremer D., Jurišić Grubešić R., Stabentheiner E., Randić M., Dunkić V. (2021). Free Volatile Compounds of *Veronica austriaca* Ssp. Jacquinii (Baumg.) Eb. Fisch. and Their Biological Activity. Plants.

[41] Mohadjerani M., Asadollahi S. (2019). *Veronica crista-galli* Steven and *Veronica persica* Poir. as Anticancer and Antioxidant Plants in-Vitro. Trends Phytochem. Res. (TPR) Trends Phytochem. Res..

[42] Saracoglu I., Oztunca F.H., Nagatsu A., Harput U.S. (2011). Iridoid Content and Biological Activities of *Veronica cuneifolia* Subsp. *Cuneifolia* and *V. Cymbalaria*. Pharm. Biol..

[43] Vrca I., Čikeš Čulić V., Lozić M., Dunkić N., Kremer D., Ruščić M., Nazlić M., Dunkić V. (2023). Isolation of Volatile Compounds by Microwave-Assisted Extraction from Six *Veronica* Species and Testing of Their Antiproliferative and Apoptotic Activities. Plants.

[44] Harput U.S., Saracoglu I., Inoue M., Ogihara Y. (2002). Anti-Inflammatory and Cytotoxic Activities of Five Veronica Species. Biol. Pharm. Bull..

[45] Moreno-Escobar J.A., Bazalda S., Villarreal M.L., Bonilla-Barbosa J.R., Mendoza S., Rodríguez-López V. (2011). Cytotoxic and Antioxidant Activities of Selected Lamiales Species from Mexico. Pharm. Biol..

[46] Feng K., Jiang R., Sun L.W. Studies on Anti-Tumor Activity in Vitro of the Flavonoid Extract in Round Leaf Speedwell. Proceedings of the First International Conference on Cellular, Molecular Biology, Biophysics and Bioengineering.

[47] Andueza A., García-Garzón A., Ruiz De Galarreta M., Ansorena E., Iraburu M.J., López-Zabalza M.J., Martínez-Irujo J.J. (2015). Oxidation Pathways Underlying the Pro-Oxidant Effects of Apigenin. Free Radic. Biol. Med..

[48] Benali T., Bakrim S., Ghchime R., Benkhaira N., El Omari N., Balahbib A., Taha D., Zengin G., Hasan M.M., Bibi S. (2024). Pharmacological Insights into the Multifaceted Biological Properties of Quinic Acid. Biotechnol. Genet. Eng. Rev..

[49] Nikolova M. (2011). Screening of Radical Scavenging Activity and Polyphenol Content of Bulgarian Plant Species. Pharmacogn. Res..

[50] Ninfali P., Mea G., Giorgini S., Rocchi M., Bacchiocca M. (2005). Antioxidant Capacity of Vegetables, Spices and Dressings Relevant to Nutrition. Br. J. Nutr..

[51] Zheng W., Wang S.Y. (2001). Antioxidant Activity and Phenolic Compounds in Selected Herbs. J. Agric. Food Chem..

[52] Wojcikowski K., Stevenson L., Leach D., Wohlmuth H., Gobe G. (2007). Antioxidant Capacity of 55 Medicinal Herbs Traditionally Used to Treat the Urinary System: A Comparison Using a Sequential Three-Solvent Extraction Process. J. Altern. Complement. Med..

[53] Horozić E., Mekić L., Cipurković S., Kolarević L., Husejnagić D., Ibišević M., Karić E., Cilović Kozarević E., Pođanin M., Brekalo-Lazarević S. (2025). Cytotoxic, Antibacterial and Antioxidant Activity of the Methanolic Extract of Speedwells (*Veronica officinalis* L.). Technol. Acta.

[54] Hassen I., Casabianca H., Hosni K. (2015). Biological Activities of the Natural Antioxidant Oleuropein: Exceeding the Expectation—A Mini-Review. J. Funct. Foods.

[55] Moghadam A., Larsen H. (2019). Importance of *Listeria monocytogenes* in Food Safety: A Review of Its Prevalence, Detection, and Antibiotic Resistance. Iran. J. Vet. Res..

[56] Kȩpa M., Miklasińska-Majdanik M., Wojtyczka R.D., Idzik D., Korzeniowski K., Smoleń-Dzirba J., Wasik T.J. (2018). Antimicrobial Potential of Caffeic Acid against *Staphylococcus aureus* Clinical Strains. BioMed Res. Int..

[57] Almuhanna Y., Alshalani A., AlSudais H., Alanazi F., Alissa M., Asad M., Joseph B. (2024). Antibacterial, Antibiofilm, and Wound Healing Activities of Rutin and Quercetin and Their Interaction with Gentamicin on Excision Wounds in Diabetic Mice. Biology.

[58] Ercan L., Dogru M. (2022). Antioxidant and Antimicrobial Capacity of Quinic Acid. Bitlis Eren Üniversitesi Fen. Bilim. Derg..

[59] Lou Z., Wang H., Rao S., Sun J., Ma C., Li J. (2012). *P*-Coumaric Acid Kills Bacteria through Dual Damage Mechanisms. Food Control.

[60] Elagdi C., Bouaouda K., Rahhal R., Hsaine M., Badri W., Fougrach H., Hajjouji H. (2023). EL Phenolic Compounds, Antioxidant and Antibacterial Activities of the Methanolic Extracts of *Euphorbia resinifera* and *Euphorbia echinus*. Sci. Afr..

[61] Darabpour E., Motamedi H., Nejad S.M.S. (2010). Antimicrobial Properties of Teucrium Polium against Some Clinical Pathogens. Asian Pac. J. Trop. Med..

[62] Dulger B., Ugurlu E. (2005). Evaluation of Antimicrobial Activity of Some Endemic Scrophulariaceae Members from Turkey. Pharm. Biol..

