# Supplementary material for: Genus Veronica—Antioxidant, Cytotoxic and Antibacterial Activity of Phenolic Compounds from Wild and Cultivated Species
Source: Antioxidants (Basel). 2025 Oct 30;14(11):1308. doi: 10.3390/antiox14111308 (PMC12649209; doi:10.3390/antiox14111308)
Supplement: Supplementary file 1 [file antioxidants-14-01308-s001.zip › antioxidants-3869703-supplementary.pdf]

Supplementary Materials

Table S1. Precursor and quantitation fragments m/z and retention time of phenolic compounds (previously included in the Vrca et al. [17])

Compound	Precursor m/z	Fragment m/z	t_r (min)
p-hydroxybenzoic acid	137.02	93.03	3.48
protocatechuic acid	153.02	109.03	2.21
Gentisic acid	153.02	109.03	3.21
Vanillic acid	167.03	152.01	4.66
Gallic acid	169.01	125.02	1.33
Syringic acid	197.04	123.01	5.59
p-coumaric acid	163.04	119.05	7.18
o-coumaric acid	163.04	119.05	9.16
Caffeic acid	179.03	135.04	4.9
Ferulic acid	193.05	134.03	8.19
Chlorogenic acid	353.08	179,03	4.64
Quinic acid	191.05	85.03	4.64
Sinapic acid	223.06	193.01	8.49
Rosmarinic acid	359.08	161.02	9.88
Cinnamic acid	147.05	103.06	11.17
Epicatechin	289.07	109.03	6.25
Catechin	289.07	109.03	4.17
Resveratrol	227.07	143.05	10.42
Astringin	405.12	243.06	7.50
EGCG (Epigallocatechin gallate)	457.08	169.01	6.95
Hesperetin	301.07	164.01	12.61
Quercetin	301.03	151.00	11.36
Myricetin	317.03	151.00	10.03
Apigenin	269.04	117.03	12.46
Naringenin	271.06	151.00	12.14
Rutin	609.15	00.03	8.86

Tables S2.-S9. Antibacterial testing of antibiotics according to EUCAST – standardized method (disc diffusion)

Table S2. *Escherichia coli* – breakpoints mm

28<br>Amoxicillin clavulanic acid 20 μg	24<br>Nitrofurantoin 100 μg
30<br>Ceftazidime 10 μg	24<br>Gentamicin 10 μg
30<br>Cefepime 30 μg	24<br>Cefuroxime 30 μg
30<br>Piperacillin + tazobactam 30 μg	26<br>Amikacin 30 μg
30<br>Ertapenem 10 μg	30<br>Ciprofloxacin 5 μg
10<br>Norfloxacin 10 μg	30<br>Imipenem 10 μg

Table S3. *Pseudomonas aeruginosa* – breakpoints mm

50<br>Piperacillin 30 µg	50<br>Cefepime 30 µg
24<br>Imipenem 10 µg	50<br>Aztreonam 30 µg
24<br>Meropenem 25 µg	CAZ -20<br>Ceftazidime 10 µg
22<br>Amikacin 30 µg	50<br>Ciprofloxacine 5 µg

Table S4. *Listeria monocytogenes*_– breakpoints mm

30<br>Amoxicillin clavulanic acid 20 µg	30<br>Tetracykline 30 µg
12<br>Cefoxitin 30 µg	30<br>Erythromycin 15 µg
30<br>Trimetoprim/sulfametoxazole 23,75 µg	30<br>Meropenem 10 µg

Table S5. *Listeria innocua*_– breakpoints mm

30<br>Amoxicillin/clavulonic acid 20 µg	30<br>Tetracykline 30 µg
20<br>Cefoxitim 30 µg	30<br>Erythromycin 15 µg
30<br>Trimetoprim/sulfametoxazol 23,75 µg	30<br>Meropenem 10 µg

Table S6. *Streptococcus pyogenes*_– breakpoints mm

30<br>Azithromycin 15µg	30<br>Erythromycin 15µg
30<br>Amoxicillin clavulanic acid 20 µg	30<br>Phenoxymethyl penicillin

Table S7. *Staphylococcus aureus*_– breakpoints mm

30<br>Linezolid 10µg	28<br>Gentamicin 10µg
30<br>Cefoxitin 30µg	26<br>Norfoxacin 10 µg
26<br>Erythromicin 15µg	30<br>Clindamycin 2µg
24<br>Linezolid 10µg	24<br>Fusidic acid 10µg
30<br>Tetracycline 30µg	20<br>Trimetoprime/sulfamethoxazole 23.75 µg

Table S8. *Enterococcus faecalis* – breakpoints mm

50<br>Piperacillin 30µg	50<br>Imipenem 10µg
24<br>Nitrofurantoine 100µg	20<br>Vankomycine 5µg
16<br>Norfloxacine 10µg	16<br>Gentamycine 30µg

Table S9. *Enterococcus faecium* – breakpoints mm

26<br>Linezolid 10 µg	18<br>Vankomycine 5 µg
16<br>Norfloxacine 10 µg	20<br>Gentamycine 30 µg
30<br>Imipenem 10 µg	20<br>Teicoplanin 30 µg
